# Pathogen and Spoilage Microorganisms in Meat and Dairy Analogues: Occurrence and Control Strategies

**DOI:** 10.3390/foods14101819

**Published:** 2025-05-20

**Authors:** José M. Martín-Miguélez, Irene Martín, Belén Peromingo, Josué Delgado, Juan J. Córdoba

**Affiliations:** Higiene y Seguridad Alimentaria, Instituto de Investigación de Carne y Productos Cárnicos (IProCar), Facultad de Veterinaria, Universidad de Extremadura, Avda. de las Ciencias s/n, 10003 Cáceres, Spain; jmmm@unex.es (J.M.M.-M.); iremartint@unex.es (I.M.); belenperomingo@unex.es (B.P.); jdperon@unex.es (J.D.)

**Keywords:** plant-based analogues, *Listeria monocytogenes*, *Salmonella*, *Clostridium botulinum*, Bacillus cereus, spore-forming bacteria, lactic acid bacteria

## Abstract

Recent advances in the production of meat and dairy analogues and plant-based products have introduced new food safety challenges, as these foods are susceptible to contamination by both pathogens and spoilage microorganisms originating from raw materials and processing environments. In addition, plant-based analogues often exhibit physicochemical properties such as high water activity, near-neutral pH, and elevated protein and moisture content that favour the survival and growth of microorganisms, as evidenced by the detection of *Salmonella* sp., *Listeria monocytogenes*, and *Enterobacteriaceae* in commercial products. While extrusion and thermal processing effectively reduce microbial loads, these treatments may not inactivate all spores, including spore-forming bacteria like *Bacillus cereus* and *Clostridium* spp. Critical findings seem to highlight that some protein isolates are particularly prone to higher microbial contamination, and that outbreaks linked to pathogens such as *Salmonella*, *L. monocytogenes*, and *E. coli* have already occurred in plant-based analogues in Europe and North America. Here we discuss the microbiology and sources of microbial contamination of these products. In addition, we further discuss the integration of non-thermal technologies and biocontrol methods, such as protective cultures, bacteriocins, and bacteriophages, as promising approaches to enhance food safety while addressing clean-label demands.

## 1. Introduction

The current trend to reduce the consumption of foods of animal origin, the projected increase in the global population, and the imperative to produce foods more sustainably have prompted the development of analogue foods made with plant-based proteins [1,2]. During the 2010s, progress in technology enabled the development of numerous alternatives to traditional meat and dairy products. Currently, the number of consumers choosing meat and dairy analogues for health reasons (such as animal protein allergies and lactose intolerance) and for their positive impact on the environment and animal welfare is increasing daily [3,4,5]. These products could be considered ultra-processed foods [6]. The possibility of contamination with pathogens usually involved in foodborne outbreaks, such as *Salmonella* spp., *Campylobacter* spp., *Listeria (L.) monocytogenes*, *Escherichia (E.) coli*, and *Staphylococcus (S.) aureus* [7], from the ingredients or during processing may pose a risk to consumers. Additionally, opportunistic microorganisms like *Cronobacter sakazakii* are increasingly recognised as a public health concern, given their capacity to contaminate diverse food ingredients and cause foodborne illnesses, which can result in mortality rates as high as 40–80% among infants and immunocompromised people [8]. Furthermore, microbial contamination in these products could represent a problem of microbial spoilage, reducing the products’ shelf life [9]. Although considerable progress has been achieved in safeguarding the microbial quality of products derived from animals, greater focus is needed on the safety of meat and dairy analogues [10,11]. These products are occasionally processed in the same facilities that manufacture meat and dairy products, and thus could be contaminated with the same pathogens or spoilage microorganisms affecting meat and dairy products [12,13]. In addition, the meat and dairy analogues usually have humidity content, water activity (a_w_), and pH that could favour microbial growth. As a result, there have already been pathogenic bacteria outbreaks in Europe and North America in plant-based analogues [14,15,16]. The hypothesis posits that the combination of near-neutral pH, high a_w_, and nutrient-rich formulations in these products creates an environment conducive to pathogenic bacteria growth, particularly for acid-tolerant pathogens like *L. monocytogenes* and *Salmonella* spp. This risk is exacerbated by the absence of traditional preservatives in clean-label formulations. Thus, it is necessary to evaluate the microbiology of these meat and dairy analogues, their sources of microbial contamination, and the conditions of these products that could favour microbial growth. Furthermore, the strategies for microbial control to produce safe meat and dairy analogues with extended shelf life and fulfilling the current trend of clean labels should be analysed. The purpose of this paper is to review the occurrence of pathogen and spoilage microorganisms in meat and dairy analogues, the sources of microbial contamination, and the strategies to control them.

## 2. Sources of Microbial Contamination of Meat and Dairy Analogues

Meat and dairy analogues are composed of various processed components and require intricate production methods, such as texturisation and extrusion, to mimic the taste, appearance, sensory characteristics, and nutritional components of animal meat [17]. The incorporation of novel plant-based ingredients into traditional products or the modification of the processing conditions of known ingredients can trigger the presence of pathogenic and spoilage microorganisms due to the greater potential for microbiological contamination through raw materials or extensive manufacturing processes [11,18].

There is a wide range of plant-based formulations used as meat and milk substitutes. Among the alternatives, plant-based proteins are the most widely used, such as proteins derived from legumes (peas, beans, chickpeas), cereals and pseudocereals (rice, oats, wheat, quinoa), nuts (almonds, cashews), and oilseeds (canola and sunflower) [19]. However, numerous uncertainties persist concerning the microbial composition associated with various plant-derived protein sources, as the baseline levels of microbial contaminants are variable and can be influenced by multiple factors, such as geographic location, climate, and growing and harvesting conditions [11]. Furthermore, plant-based ingredients originate from diverse sources and are available in multiple forms, including concentrated liquids or pastes, powdered forms (concentrates and isolates), as well as flours made from whole seeds, nuts, or cereals. Each of these formats exhibits distinct degrees of purity and protein concentration, factors that can affect both the quantity and variety of microorganisms present [11]. Depending on the raw materials selected, high microbial loads, including pathogenic species, can occur in initial formulations and premixes.

The main microbiological contamination occurs through raw materials, primarily dry protein powders. The low a_w_ of these raw materials does not promote the growth of microorganisms, but several bacterial and endospore-forming species can survive these conditions [20]. By adding water to plant-based dry protein powder before processing, an initial microbiota derived from the raw material and/or the production environment can emerge and multiply, though bacterial species and survival times could affect the microbial load of the rehydrated product [21,22]. Furthermore, the lack of hygienic equipment design and poor water hygiene pose a contamination risk to the intermediate and final protein matrix [23]. One of the main microbial groups found in raw materials is spore-forming microorganisms such as *Bacillus* spp. and *Clostridium* spp. The ability to form endospores allows *Bacillus* and *Clostridium* species to survive in many diverse environments [24]. Furthermore, numerous *Bacillus* species are widely distributed in soil environments and act as plant-growth-promoting bacteria, thereby enhancing soil health and fertility [25,26]. As a consequence, *Bacillus* species are commonly found in a wide range of foods, particularly those derived from plants. Furthermore, *Bacillus cereus* is capable of colonising food processing equipment and developing biofilms, which may subsequently serve as a source of contamination for final food products [27]. To avoid these problems, food manufacturers employ various food inactivation and preservation strategies. However, applying thermal processes to destroy spores, as commonly used in dairy products, may not be appropriate for plant-based dairy alternatives. This is due to differences in the chemical and physical characteristics of plant-derived ingredients in comparison to those of milk (e.g., differences in protein solubility/precipitation or substrate availability) [11]. On the other hand, in plant-based meat analogues, heat-stable spore-forming microorganisms could survive extrusion cooking and germinate under storage conditions. Therefore, it is important to thoroughly examine intermediate products for survival, spoilage, and potentially pathogenic and toxigenic microorganisms. In the EU-commissioned “LikeMeat” research project, the presence of spoilage microorganisms, potential pathogens, heat-stable microorganisms, and spore-forming microorganisms was examined in both different protein raw materials and intermediate products processed by extrusion. Overall, among the raw materials examined, lupin protein isolate showed an average mesophilic aerobic count of 2.4 × 10^4^ CFU/g, the highest contamination with *Enterobacteriaceae* and coliforms was detected in pea protein isolate (1.1 × 10^3^ CFU/g and 4.6 × 10^2^ CFU/g, respectively), and the presence of *Bacillus* spp. was detected in pea protein isolate (2.6 × 10^2^ CFU/g) and lupin protein isolate (9 × 10^2^ CFU/g). However, foodborne pathogens, including *Salmonella* species, *L. monocytogenes*, *Clostridium* species, and *B. cereus*, tested negative in all samples analysed. On the other hand, the microbiological quality of the extruded products was satisfactory in all samples analysed. This suggests that all microorganisms monitored in the raw material did not survive the extrusion cooking process [23]. However, a study by Kyrylenko et al. (2023) [11] detected high levels of *Bacillus stearothermophilus* in pea isolates used in dairy alternatives due to defective processing conditions, highlighting the importance of processing control to prevent contamination.

Plant-based meat substitutes typically possess elevated protein and moisture contents along with a neutral pH, factors that collectively promote the proliferation of spoilage microbes and foodborne pathogens [28,29,30]. This increases the possibility of recontamination of plant-based analogues after processing and during storage of the final product, affecting the shelf life of the final product. The recontamination of these products prior to commercialisation, such as slicing and packaging, has been reported as a potential safety concern [31]. These authors found *Listeria* spp. in meat analogues due to recontamination. This recontamination was reinforced by the identification of *Lactobacillus sakei*, *Enterococcus faecium*, and *Carnobacterium divergens* in plant-based meat analogues [32]. A similar study revealed significant amounts of *Enterobacteriaceae* and yeast species during storage in both refrigerated and unrefrigerated meat analogues [9]. Similarly, a recent study on both pea-based and soybean-based plant-based meat analogues suggested that they could support the persistence and potentially the proliferation of spoilage microorganisms, such as *Pseudomonas fluorescens* and *Brochothrix thermosphacta*, as well as pathogens such as *E. coli*, *Salmonella* spp., and *L. monocytogenes* [29]. Therefore, all post-processing steps of plant-based analogues could be a main source of microbial contamination of these products, considering that many of these microorganisms, such as *L. monocytogenes* can form biofilms [33]. The risk of recontamination arising from product handling and packaging should be minimised. Therefore, Good Hygienic Practices (GHP) should be maintained to ensure that recontamination does not occur, and different thermal and non-thermal post-packaging treatments of the final product should be considered.

## 3. Microbiology of Meat and Dairy Analogues

The microbial population found in animal-derived products such as milk or meat is completely different from the predominant one in plant-based ingredients [34,35]. Animal-derived products with high microbial loads of coliforms are typically linked to inadequate hygiene during food processing, serving as critical indicators of potential contamination by pathogenic genera such as *Salmonella*, *E. coli*, *Shigella*, and *Yersinia* [36]. This poses significant risks, as the presence of *Enterobacteriaceae* often signals improper sanitation, post-processing contamination (e.g., via equipment or handlers), or insufficient thermal treatment [37]. In contrast, the main microbial population in plant-based ingredients consists of spore formers such as *Bacillus* spp. and *Clostridium* spp. [11]. These spores are particularly challenging to eliminate due to their resilience to heat and other environmental stresses, posing a risk to food safety if not properly managed [38].

The evaluation under identical conditions of meat products and meat analogues unveils the microbial differences between both types of foods to execute preventive actions appropriate for each case [39]. This understanding is crucial for implementing targeted preventive measures tailored to each type of product. The presence of pathogens and spoilage microorganisms during the processing of meat and dairy analogues might imply a safety issue for consumers. Thus, continuous oversight and management of microbial contamination at every stage of the production process are crucial to guarantee both the safety and quality of these products.

### 3.1. Pathogen Microorganisms

Plant-based meat analogues typically undergo thermal treatment to ensure self-stability during the commercialisation process and achieve a meat-like texture [40]. The extrusion process, a common method used in plant-based meat production, involves the application of heat and mechanical energy to transform plant proteins into a meat-like texture [41]. However, this process may not be effective against spore-forming bacteria such as *B. cereus* and *Clostridium perfringens*, which can survive the heating step and pose a safety concern if not properly managed [42].

Similarly, like dairy products, dairy analogues also undergo pasteurisation or sterilisation to reduce perishability and improve technological properties such as stability [43,44]. The pursuit of improved texturing through non-thermomechanical processing methods may introduce microbiological safety concerns [45]. Spore formers are known to be identified in pulses, cereals, and drupes, the ingredients of plant-based analogues [11].

The development of safer processing methods for meat and dairy analogues is a key research objective globally [46]. The knowledge of the pathogenic bacteria that can develop on plant-based analogues and their differentiation from foods of animal origin that they try to mimic is essential to increase awareness and apply effective control strategies against their development. Table 1 lists pathogenic bacteria that have been previously isolated in commercialised plant-based meat analogues.

Few studies have reported on pathogenic microorganisms found in commercialised plant-based dairy analogues. Some pathogenic bacteria have been found in plant-based cheese analogues, as has been proven by the recorded foodborne diseases in Ready-To-Eat (RTE) plant-based analogues [14,15,16]. Ensuring the microbiological safety of plant-based analogues requires a comprehensive approach that includes proper processing techniques, storage conditions, and handling practices. As consumer demand for these products continues to grow, addressing these safety concerns is crucial to maintain consumer trust and prevent foodborne illnesses. Furthermore, consumer education on the hazards of RTE plant-based analogues is necessary, as they can have the same risks as RTE products of animal origin.

### 3.2. Spoilage Microorganisms

The higher presence of aerobic microorganisms responsible for the spoilage of plant-based meat analogues seems to reduce their shelf life [9]. However, aerobic and anaerobic microorganisms such as *Enterobacteriaceae*, lactic acid bacteria (LAB), and yeasts have been reported as spoilage microorganisms in plant-based analogues (Table 2).

*Enterobacteriaceae* are often associated with raw vegetables and can survive processing steps if not properly controlled [36]. In plant-based meat analogues, *Enterobacteriaceae* have been detected, particularly in products made from pea protein, indicating that these bacteria can persist in certain types of raw materials [39].

LAB have been proven to become spoilage agents in thermally treated analogues, as they can be naturally present in the ingredients used in these products [11]. LAB presence can indicate contamination if they are not part of the intended microbial culture [50]. Thus, fermented products such as cheese or ripened meat analogues are usually produced with LAB [51,52,53]. When intentionally used, LAB are beneficial for food safety and quality, and high microbial counts are found during the shelf life of the product, making it difficult to know if contamination with another strain of this microbial group has occurred [49]. However, LAB are well known to be a protective agent in a wide variety of products, and they also ensure safety in plant-based fermented products [54,55].

Yeasts, while not as commonly discussed in the context of plant-based analogues, can also be present due to contamination during processing [9,42]. Yeasts are generally more associated with fermented products, where they contribute to flavour and texture, but they can also be spoilage agents if not controlled [56]. The presence of yeasts in plant-based products might be less common compared to bacteria, but can still pose a risk if the products are not stored properly [9,57].

To the best of the authors’ knowledge, there is a lack of literature in the field of spoilage microorganisms in commercialised plant-based dairy analogues. The study of spoilage agents in dairy analogues should be intensified to guarantee the application of correct hygiene measures and higher protection of consumers’ safety. The thermal processing through which plant-based dairy analogues usually undergo can mitigate the hazards of these products, but vegetal raw materials can allow the development of spoilage and pathogenic microorganisms, as it may happen in the animal products they try to mimic [58,59].

The protein source and its relationship with the food safety of the product should also be more intensely researched, as pea protein has been proven by other authors to be more microbiologically compromised than soy protein [39]. However, there is a current lack of research in this area, and further investigation should be performed to understand whether pea protein contamination could have taken place in previous studies or whether this raw material is indeed more microbiologically compromised. The microbial susceptibility of each protein source should be well known by industries and consumers, as the allergenicity of plant-based proteins is being widely studied [60,61].

## 4. Behaviour of Pathogens and Spoilage Microorganisms in Food Analogues

The physicochemical characteristics of the meat and dairy analogues, together with the conditions in which these foods are stored, determine microbial development and behaviour.

Both survival and growth of pathogens such as *E. coli* HEHA16, *L. monocytogenes*, *Salmonella enterica* ser. Typhi, and *C. sakazakii* [2,29] and spoilage microorganisms such as *P. fluorescens* and *B. thermosphacta* [29] have been proven in meat analogues. The behaviour of these pathogens is influenced by pH, a_w_, storage temperature, and the presence of preservatives in the formulation of food analogues, as we discuss below.

### 4.1. pH

The pH of the medium significantly affects microbial growth and even virulence, as previously stated [62], where a marked elevation in the relative expression levels of virulence genes was observed in the most acidic environments tested, concretely at pH 4.5. Most microorganisms thrive in a neutral pH environment, although some can tolerate or even require acidic or alkaline conditions. For instance, LAB contribute to acidification, while yeasts can tolerate a broader pH range. In plant-based meat analogues, the pH can vary depending on the protein source used. After examining pH data from the two main plant-based protein sources, it seems that there is no clear relationship between soy-based meat analogues or pea-based ones with pH values [29,42], which influence microbial growth. Moreover, the pH of the product is modified along with storage conditions as a consequence of the physicochemical reactions, and, more importantly, because of the microorganism’s physiology. In a study assessing the initial pH of pea-based analogues, it was around 7.38, and decreased to 6.26 over 10 days of storage at 4 °C, and for soy-based ones, it was 6.16, with almost no modification over the study period, ending at 6.22 [29]. Additionally, these values were not correlated with LAB development. This is likely related to the type of LAB that originally contaminated the food matrix, since every LAB species has a different ability to decrease the pH of the food [63].

### 4.2. Water Activity

The a_w_ is another critical factor affecting microbial growth. Meat analogues generally have high a_w_ [42], making them susceptible to microbial spoilage. High a_w_ values allow microorganisms to access water for metabolic processes, facilitating growth and toxin production. Some values gathered among 43 samples from the Czech Republic showed a_w_ values between 0.959 and 0.977. Regarding plant-based yoghurt analogues from sources such as soy, almond, coconut, cashew, and oat, the a_w_ varied between 0.97 and 0.99 [64], whilst plant-based cheese analogues made from mainly coconut oil and different starches showed a_w_ values from 0.95 to 0.99 [65]. These a_w_ values could be considered high since it is not possible to inhibit most of the pathogen and spoilage microorganisms with only this parameter.

### 4.3. Temperature

Temperature is a critical environmental factor affecting microbial growth in meat and dairy analogues. Each microorganism has an optimal temperature range for growth, and temperatures outside this range can inhibit or kill them, as we discuss in Section 5.1. *L. monocytogenes* can grow at refrigeration temperatures, especially in pea-based meat analogues, where they increased by about 0.74 log CFU/g during 7-day storage at 4 °C [29]. Furthermore, this study showed that at abused temperatures, pathogens like *Salmonella* spp. and *E. coli* O157:H7 were able to grow rapidly in both meat products and meat analogues.

Furthermore, studies using metagenomic sequencing have shown that plant-based meat analogues can support the survival and growth of foodborne pathogens like *L. monocytogenes* and *C. sakazakii*, especially in matrices with favourable conditions such as high moisture and neutral pH [2]. This underscores the importance of understanding how different matrices influence pathogen dynamics.

### 4.4. Preservative

Preservatives added to food analogues can significantly inhibit microbial growth. Therefore, preservatives can extend shelf life by preventing the growth of spoilage microorganisms. Although natural compounds could be used as preservatives, many of them present disadvantages, such as sensory impact, or they are not as powerful, or they are only effective on some types of microorganisms, as is the case of sorbic acid and benzoic acid that are effective mainly against yeasts and bacteria, respectively [66,67]. However, the use of these common preservatives is not desired by consumers since the current tendency is the clean label [68,69]. Therefore, there is a critical need for clean and low-environmental-impact strategies to enhance the food safety and the shelf life of these products, as discussed in Section 5.2.

Thus, pathogen and/or spoilage microorganisms can reach the food analogues by contamination mainly through the raw materials or by subsequent recontamination during processing. The contaminating microorganisms could even grow in this matrix due to the pH and a_w_ conditions of these products. Microbial control methods are needed to eliminate the risk of infection or food poisoning and to increase shelf life.

## 5. Control of Pathogens and Spoilage Microorganisms

### 5.1. Thermal and Non-Thermal Technologies

Conventional thermal food preservation technologies expose meat and dairy analogues to a high temperature, which is effective in reducing microbial contamination, but results in some undesirable changes in nutritional and sensorial characteristics [70]. Sterilisation and pasteurisation of these analogues increase their shelf life, but some negative effects may occur due to the heat treatment, such as modification of the structure, a decrease in particle size, alteration of the lipid fraction, and changes in the colour and aroma of the final products [71,72]. Novel thermal technologies such as ohmic, microwave, and ratio heating have been developed to improve the effectiveness of thermal processing in reducing microbial load in foods [58], with minimal effect on the structural and sensory characteristics of the products. These methods could be applied to reduce microbial contamination in meat and dairy analogues, especially in post-processing, such as slicing and packaging. However, in most cases, it is necessary to address the combined use of thermal processing with emerging non-thermal technologies such as pulsed electric fields and high hydrostatic pressure to complement the microbicidal effect of thermal processing [73].

Non-thermal technologies such as high hydrostatic pressure (HHP), pulsed electric field, ultrasound, ultraviolet irradiation (UV), and electron beam (E-beam) may be alternatives to thermal preservation to reduce microbial contamination in meat and dairy analogues [46], especially in sliced and packaged products. HHP is used in these products with the aim of both extending shelf life and improving features such as homogenisation, physical stability, and extraction of the compound of interest from the raw material [74,75] The pulsed electric field has been reported to be efficient in reducing microbial load in food analogues without compromising their sensory and nutritional properties [72,76]. Ultrasound, as an emerging physical technology, has demonstrated efficacy in reducing *L. monocytogenes* and *E. coli* O157:H7, although further optimisation is needed for the application of food analogues [77]. E-beam using low-dose ionising radiation has been proven to be highly effective in reducing pathogens such as *Salmonella*, *L. monocytogenes*, and *E. coli* O157:H7 in different foods [78,79], and could be applied to ensure food safety and increase shelf life in analogues. UV light technology at 254 nm is an environmentally friendly and economical treatment that has shown effectiveness in reducing pathogens such as *S. enterica* ser. Enteritidis, *B. cereus*, and *L. monocytogenes* and spoilage microorganisms on the surface of food products, including plant-based foods [80,81], and could be of great interest for the reduction or elimination of microbial contamination in the slicing of meat and dairy analogues. In addition, the combination of some of the above non-thermal technologies could be very effective for the reduction or elimination of microbial contamination in food analogues, which would result in improvements in their safety and shelf life [82,83,84].

### 5.2. Biocontrol in Meat and Dairy Analogues

Biocontrol strategies, which involve the use of natural or controlled microbiota and their antimicrobial products, are gaining prominence in ensuring the safety and quality of meat and dairy analogues [85]. These strategies are particularly relevant given the rising consumer demand for plant-based alternatives and the need to address potential microbial contamination in these products [86]. Biocontrol offers a natural and effective alternative to enhance the microbial safety of these products, including the use of protective cultures, bacteriocins, bacteriophages, and active packaging solutions, to mitigate the risks posed by pathogens and spoilage microorganisms in meat and dairy analogues [86].

#### 5.2.1. Application of LAB and Their Metabolites

LAB, generally considered safe for consumption, are well known for their ability to inhibit microbial growth, mainly due to their synthesis of organic acids, bacteriocins, and other antimicrobial substances. Their application in biocontrol as protective cultures is extended in traditional meat and dairy products and holds potential for plant-based analogues.

- Protective Cultures: LAB have been extensively studied for their antagonistic properties against foodborne pathogens. The incorporation of LAB such as *Latilactobacillus sakei*, *Lacticaseibacillus casei*, *Lactiplantibacillus plantarum*, and *Leuconostoc mesenteroides*, among others, as protective cultures, all of them with a qualified presumption of safety (QPS) [87], have demonstrated their ability to inhibit pathogenic bacteria such as *L. monocytogenes*, *Salmonella*, and *E. coli* O157:H7 and spoilage microorganisms such as *Pseudomonas* and *Enterobacteriaceae* in food products [88], obtaining reductions in *L. monocytogenes* up to 2.2 log CFU/g in dry cured fermented sausages and up to 5 log CFU/g in ripened cheeses [89,90]. Incorporating LAB into plant-based products can inhibit undesirable microorganisms through competitive exclusion and the production of antimicrobial metabolites. Recent investigations have shown the effectiveness of LAB in extending the shelf life and enhancing the safety of meat analogues [53,91,92,93].
- Bacteriocins: These are ribosomally synthesised antimicrobial peptides produced by LAB that exhibit activity against a range of pathogens, including *L. monocytogenes*. For instance, bacteriocin-producing strains have been effective in reducing *L. monocytogenes* in RTE meat and dairy products [94]. Applying similar strategies to plant-based meats could enhance their safety profile. So, incorporating bacteriocins into plant-based meat products could significantly reduce microbial load without adversely affecting sensory attributes.

#### 5.2.2. Bacteriophage Applications

Bacteriophages, viruses that target and destroy bacterial cells, have been explored as biocontrol agents in food systems. Commercial phage products targeting *L. monocytogenes* have shown efficacy in reducing pathogen levels (until 2 log CFU/g) in meat products and soft cheese, and those targeting *Salmonella* have experienced a ~3.5 log CFU/g decrease at 5 and 10 °C in apple slices [95]. Their application in food systems, including meat and dairy analogues, has shown promise in reducing contaminants such as *L. monocytogenes* and *E. coli*. Recent research has demonstrated that incorporating phage-based strategies within hurdle technology frameworks can significantly improve food safety. Investigating the application of such phages in meat and dairy analogues could offer an additional layer of safety [96]. In comparison with the LAB activity previously discussed, bacteriophages often provide high specificity and rapid action against targeted pathogens, whereas LAB can exert broader antimicrobial activity and enhance food preservation through natural fermentation processes. In complex microbial environments, a synergistic application of both LAB and bacteriophage may offer improved pathogen control and overall food safety [97,98,99].

#### 5.2.3. Active Packaging Solutions

Advancements in packaging technologies have resulted in the development of active packaging solutions that include antimicrobial agents, a promising method for preventing microbial contamination and spoilage during food distribution. These systems work by embedding antimicrobial agents into packaging materials, allowing gradual release or absorption onto the food surface, where contamination typically begins, thereby prolonging the shelf life and preserving the quality of the product [100]. This method avoids the direct addition of preservatives to food, adapting to consumer preferences [101]. Nanomaterials improve antimicrobial packaging thanks to their large surface area, reactivity, and efficacy [102]. A wide range of antimicrobial substances have been used, including inorganic (e.g., silver, ZnO), organic (e.g., enzymes, acids), natural extracts (e.g., essential oils, plant extracts), peptides (e.g., nisin), and other bioactive compounds (e.g., carvacrol, thymol) [103,104,105]. The integration of natural antimicrobial agents, including essential oils, into packaging systems has been explored to inhibit microbial growth in plant-based meat products [106,107].

## 6. Conclusions and Future Remarks

One of the main challenges in managing microbial contamination in meat and dairy analogues is the lack of homogeneity in their physicochemical characteristics. These products vary widely in terms of pH, water activity, and moisture content, which can influence microbial growth and survival. For instance, plant-based meat analogues typically have high moisture levels and a neutral pH, creating favourable conditions for microbial growth. This variability necessitates a case-by-case approach to understanding and managing microbial risks, as strategies effective for one type of product may not be suitable for another.

Given the complexity and variability of meat and dairy analogues, there is a clear requirement for detailed, product-specific studies. Each product’s unique physicochemical properties and processing conditions must be considered to develop targeted strategies for microbial control. This tailored approach will help ensure that plant-based analogues meet consumer expectations for safety and quality while aligning with the trend toward clean labels and sustainable food production.

Looking forward, new promising strategies are emerging to enhance the microbial safety and shelf life of meat and dairy analogues. The combination of non-thermal technologies with biocontrol methods, including the use of protective cultures, bacteriocins, bacteriophages, and active packaging solutions, offers natural and effective alternatives to traditional preservation techniques. These approaches not only address consumer preferences for clean-label products but also provide innovative solutions to mitigate the risks posed by pathogens and spoilage microorganisms. Given the increasing interest in plant-based analogues, further research into these strategies will be crucial for maintaining consumer trust and ensuring the long-term viability of the meat and dairy analogue market.

## Figures and Tables

**Table 1:** Pathogenic bacteria isolated from commercialised plant-based meat analogues.

Plant-Based Meat Analogue	Protein Source	Pathogenic Bacteria	Source
Ground meat	Soy, pea	*Escherichia coli* O157:H7 *Salmonella* spp. *Listeria monocytogenes*	[29]
Burger, steak, meatball	Soy, pea, wheat	*Bacillus cereus*	[42]
Ground meat	Soy, pea	*Escherichia coli* HEHA16 *Listeria monocytogenes* *Salmonella enterica* ser. Typhi *Cronobacter sakazakii*	[2]
Fermented sausage, sausage, meatball	Pulses	*Salmonella* spp. *Listeria monocytogenes*	[47]

**Table 2:** Spoilage microorganisms isolated from commercialised plant-based meat analogues.

Plant-Based Analogue	Protein Source	Spoilage Microorganisms	Source
Ground meat	Soy, pea	*Pseudomonas fluorescens* *Brochothrix thermosphacta*	[29]
Burger, steak, meatball	Soy, pea, wheat	Lactic acid bacteria *Enterobacteriaceae* Yeasts	[42]
Burger, steak, sausages	Soy, pea	Lactic acid bacteria	[48]
Sausage	Soy	Lactic acid bacteria *Thermoactinomyces* spp.	[10]
Ground meat	Soy, pea	*Brochothrix* spp. Lactic acid bacteria	[39]
Soft ripened cheese	Cashew nut	*Pediococcus pentosaceus* *Enterococcus* spp.	[49]

## Data Availability

No new data were created or analysed in this study. Data sharing is not applicable to this article.
